# Patterns in the sequential treatment of rheumatoid arthritis patients starting a b/tsDMARD: 10-year experience from a US-based registry

**DOI:** 10.21203/rs.3.rs-2624931/v1

**Published:** 2023-02-27

**Authors:** Anton Matsson, Daniel H. Solomon, Margaux M. Crabtree, Ryan W. Harrison, Heather J. Litman, Fredrik D. Johansson

**Affiliations:** Chalmers University of Technology

**Keywords:** rheumatoid arthritis, b/tsDMARDs, treatment patterns

## Abstract

**Objectives:**

Developing and evaluating new treatment guidelines for rheumatoid arthritis (RA) based on observational data requires a quantitative understanding of patterns in current treatment practice with biologic and targeted synthetic disease-modifying anti-rheumatic drugs (b/tsDMARDs).

**Methods:**

We used data from the CorEvitas RA registry to study patients starting their first b/tsDMARD therapy—defined as the first line of therapy—between 2012 and the end of 2021. We identified treatment patterns as unique sequences of therapy changes following and including the first-line therapy. Therapy cycling was defined as switching back to a treatment from a previously used therapeutic class.

**Results:**

6,015 b/tsDMARD-naive patients (77% female) were included in the analysis. Their median age was 58 years, and their median disease duration was 3 years. In 2012–2014, 80% of the patients started a tumor necrosis factor inhibitor (TNFi) as their first b/tsDMARD. However, the use of TNFi decreased in favour of Janus kinase inhibitors (JAKi) since 2015. While the number of treatment patterns was large, therapy cycling was relatively common. For example, 601 patterns were observed among 1133 patients who changed therapy at least four times, of whom 85.3% experienced therapy cycling. Furthermore, the duration of each of the first three lines of therapy decreased over the past decade.

**Conclusion:**

First-line therapy was almost always TNFi, but diversity in treatment choice was high after that. This practice variation allows for proposing and evaluating new guidelines for sequential treatment of RA. It also presents statistical challenges to compare subjects with different treatment sequences.

## Introduction

The treatment of rheumatoid arthritis (RA) patients with disease-modifying anti-rheumatic drugs (DMARDs) is often sequential and requires trial and error. While prescribing conventional synthetic DMARDs (csDMARDs) is the recommended first treatment strategy (1, 2), there is no consensus on how to choose from biologic and targeted synthetic DMARDs (b/tsDMARDs) when the initial csDMARD therapy fails. Tumor necrosis factor inhibitors (TNFi), a group of bDMARDs developed in the late 1990s, are routinely used after initial csDMARD failure (3, 4); however, current guidelines do not express any preference of bDMARDs over tsDMARDs in this situation (1, 2). With a growing number of medications available, practice variation in RA treatment continues to increase.

Implementing active control trials of sequential treatment strategies is difficult but would help with an evidence basis for stronger treatment guidelines. As an alternative, observational data on treatment decisions and outcomes might provide opportunities to evaluate new strategies without requiring the expenses and/or prolonged duration of randomized controlled trials (5). For such an evaluation to be most useful, it is necessary that alternative treatment strategies of interest are regularly observed in routine data and thus can be assessed using real-world evidence (6). Characterizing common treatment sequences is therefore an important step in finding optimal strategies for the sequential treatment of RA.

Much attention has been given to patterns in the treatment of RA patients who experience an insufficient response to their first TNFi after already having tried a csDMARD (7, 8). These patients may be treated with either a second TNFi or a medication with a new mechanism of action (9–11). However, these studies are mostly limited to the choice of second-line b/tsDMARD and do not give a complete picture of current practice. Other studies have extended the duration of follow-up and examined sequential therapies using observational designs (12, 13). Most often, these studies demonstrate transitions between therapies in a flow diagram—typically in the form of a Sankey diagram—and not the whole sequences of treatments. For example, a transition from a TNFi to a non-TNFi b/tsDMARD appears the same in a Sankey diagram regardless of previous treatments. A different set of studies have focused on pathways to a particular type of therapy, for example tocilizumab monotherapy (14) or therapies with baricitinib (15), but they do not summarize the entire treatment strategy across the disease course.

In the current set of analyses, we aimed to provide a more complete description of common patterns in the sequential treatment of RA. Using data from the CorEvitas RA registry (16), we defined the first b/tsDMARD therapy as the first line of therapy. We then described first-line therapy selection and the most common patterns of sequential therapies. In addition, given the increase in the number of available DMARDs over the past decade, we studied changes in these patterns over time. Insights from these analyses give directions for using observational data in evaluating sequential therapies for RA.

## Methods

### Study design and population

We used data from patients enrolled in the CorEvitas RA registry (16)—an ongoing longitudinal clinical registry in the US—between January 2012 and December 2021. As of March 31, 2022, data on 58,337 patients with RA were collected in the registry and include 458,982 patient visits and 228,871 patient-years of follow-up observation time. The mean duration of patient follow-up is 4.8 years (median 3.4). In addition to treatment changes and treatment history, the data include for example patient demographics, clinical disease characteristics, comorbidities, infections, and adverse events.

The aim of this study was to describe patterns in treatment sequences starting with the first b/tsDMARD therapy. We therefore selected a cohort of b/tsDMARD-naive patients who initiated a b/tsDMARD treatment at or after enrolment in the registry. The visit of the first reported b/tsDMARD initiation was considered the baseline visit and subsequent visits were defined as the follow-up period. No restrictions, for example in terms of regularity, were placed on the follow-up visits, although the registry protocol recommends visits every six months per clinical practice. In the selected cohort, 29% of the patients had at least one registry visit every 6-month period starting with the baseline visit and 75% had at least one registry visit every 12-month period. Furthermore, each patient was followed until their last recorded visit, or the data cut date of 12/31/2021 (whichever occurred first), resulting in the number of registry visits and duration of follow-up varying across patients.

### Classes of drugs and therapies

Over 20 individual drugs were at some point prescribed in the selected data. To limit the number of treatment patterns (see below), we studied the following classes of drugs rather than individual drugs: csDMARDs (hydroxychloroquine, leflunomide, methotrexate, sulfasalazine, cyclosporine, azathioprine, and minocycline hydrochloride), TNFi (adalimumab, certolizumab pegol, etanercept, golimumab, infliximab, and biosimilar TNFi), interleukin-6 receptor inhibitors (IL-6Ri) (sarilumab and tocilizumab), T-cell inhibitors (abatacept), B-cell inhibitors (rituximab), and Janus kinase inhibitors (JAKi) (baricitinib, tofacitinib, and upadacitinib). Interleukin-1 receptor inhibitors were excluded due to small sample size. For the sake of simplicity, we refer to abatacept and rituximab instead of T-cell inhibitors and B-cell inhibitors below.

Based on these drug classes, we labelled prescribed therapies for each patient in the data. We included both monotherapies and combination therapies with potentially multiple csDMARDs in combination with one b/tsDMARD. We did not distinguish between csDMARD monotherapies and csDMARD-only combination therapies. For example, both a methotrexate monotherapy and a methotrexate plus leflunomide combination therapy were labelled a csDMARD therapy. Furthermore, we always highlighted the use of a b/tsDMARD. For example, a therapy with csDMARDs in combination with TNFi was considered a TNFi combination therapy, regardless of which drug was added last and which csDMARDs were used. A therapy without any DMARDs was labelled “No DMARD”. We did not categorize combinations of b/tsDMARDs, e.g., TNFi in combination with JAKi, as they are not recommended clinically. Such therapies were rare (less than 1 % of all prescribed therapies) and classified as “Other”.

In summary, we studied the following classes of therapies: csDMARD therapy, TNFi monotherapy, TNFi combination therapy, IL-6Ri monotherapy, IL-6Ri combination therapy, abatacept monotherapy, abatacept combination therapy, rituximab monotherapy, rituximab combination therapy, JAKi monotherapy, JAKi combination therapy, and no DMARD therapy. The initial b/tsDMARD therapy was defined as the first line of therapy.

### Treatment patterns

We defined a treatment pattern of length *m* as a *unique* sequence consisting of a first-line therapy and the *m* − 1 *therapy changes* following the first-line therapy. For example, given an initial TNFi combination therapy, replacing the TNFi with a JAKi means a change of therapy to a JAKi combination therapy, and stopping all csDMARDs gives a TNFi monotherapy. While a sequence may refer to *m* arbitrarily consecutive therapies, a pattern is a particular sequence, for example, with *m* = 3, “TNFi combo → TNFi mono → No DMARD”. Cycling between drugs within the same drug class, for example replacing etanercept with adalimumab in a TNFi monotherapy, is not considered a therapy change with our definition of therapy classes. This choice was made for statistical reasons to limit the number of possible treatment patterns. We defined *therapy cycling* as returning to a previously used therapy class.

For a fixed sequence length *m*, we collected all patients in the selected cohort who changed therapy at least *m* − 1 times from baseline and onwards. We counted the number of occurrences of each unique therapy sequence—each pattern—and created visualizations of the most common ones. For *m* = 1, i.e., baseline, we summarized the most common treatments using bar plots. For a given *m* ≥ 3, we presented the most frequent patterns in a single figure. To make this visualization as informative as possible, we grouped similar patterns together, and we set the length of each therapy segment to the median duration of that therapy in the data.

### Statistical analysis

Patient characteristics at baseline were summarized with descriptive statistics. Categorical variables were summarized using frequency counts and percentages; continuous variables were summarized by median, first quartile and third quartile.

Selected results were studied from a time perspective. Specifically, we divided the data into three distinct groups based on the date of the *baseline visit*. 2012 to 2014, 2015 to 2017, and 2018 to 2021. The number of groups was limited to three to ensure that all groups contained sufficiently many patients, and the intervals were chosen to be almost evenly distributed. Follow-up visits were allowed to occur beyond the distinct calendar year groupings. When studying the duration of the *m*th therapy across time periods, we included only patients who were treated with at least *m* + 1 therapies to avoid censoring. We recognise that this requirement introduced bias since patients who remained on the *m*th therapy were excluded. Note, however, that requiring a minimum follow-up duration for patients in the cohort would not resolve this issue since patients change therapy at different frequencies. We compared distribution medians using Kruskal-Wallis H-tests with a significance level of *a* = 0.001.

We used Python version 3.10 to conduct the data analyses. The data were processed using Pandas (17) and NumPy (18), figures were created using Matplotlib (19) and Seaborn (20), and statistical tests were performed using SciPy (21).

## Results

### Baseline characteristics

We identified 6,015 unique patients (77% female) who met the study criteria. Of the 40,882 patients in the original dataset, 21,507 (53%) had a history of b/tsDMARD usage at the first visit registered in the data, and 13,360 (33%) never started a b/tsDMARD treatment. At baseline, the median age of the selected patients was 58 years, and the median disease duration was 3 years. Additional baseline characteristics are given in Table 1.

### First-line therapy selection over the past decade

**Figure 1:**
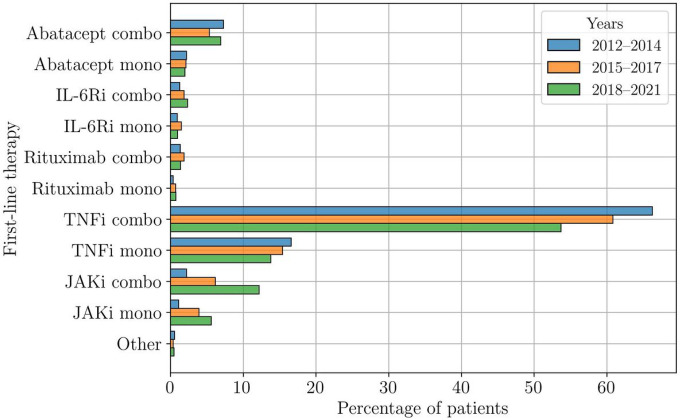
Distribution of first-line therapies for different time periods in the last decade. Up to 80% of the patients started a TNFi as their first b/tsDMARD, either as monotherapy or in combination with csDMARDs. However, in recent years, the use of TNFi decreased, while the use of JAKi increased. The use of the other drugs has remained relatively constant.

Figure 1 shows the distribution of the first b/tsDMARD therapy for different time periods in the last decade. Therapies with TNFi dominated—up to 80% of the patients received either a TNFi monotherapy or TNFi in combination with csDMARDs—but the use of JAKi steadily increased. In the most recent interval, almost 20% of the patients were treated with JAKi therapies as their first b/tsDMARD therapy. This change appears concurrent with a reduction of TNFi prescriptions; the use of other therapies remained relatively constant.

### From consensus to heterogeneity

**Figure 2:**
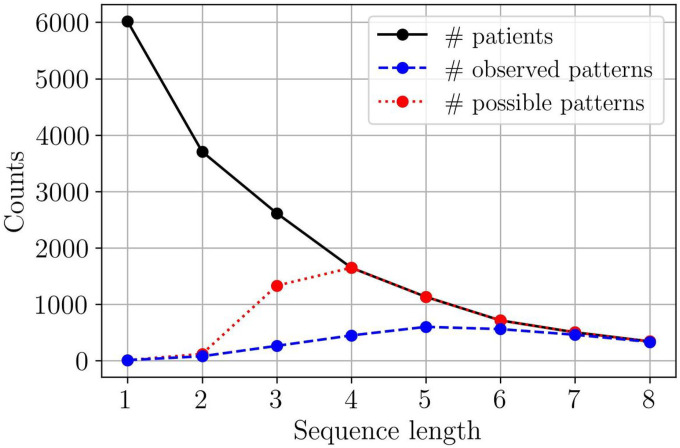
Number of patients and patterns against sequence length. The number of patients treated with at least *m-1* therapies following the baseline therapy(solid black line) and the number of observed patterns (dashed blue line) as a function of *m*, the number of therapies in sequence. The number of possible patterns (dotted red line) is included as a reference. Recall that a pattern is defined as a unique therapy sequence.

With the aim of describing patterns in current treatment practice, we studied the ratio between the number of patients and the number of observed patterns. Figure 2 shows how this ratio varies with the number of therapies in sequence. For example, all 6,015 patients in the selected cohort were treated with at least one therapy, but only one sixth of the patients were treated with five therapies or more. The number of observed patterns increases from 11 to 601, approaching the maximum number of patterns. In other words, there were fewer *recurring patterns* for longer sequences.

### Patterns in the first three to five lines of therapy

**Figure 3:**
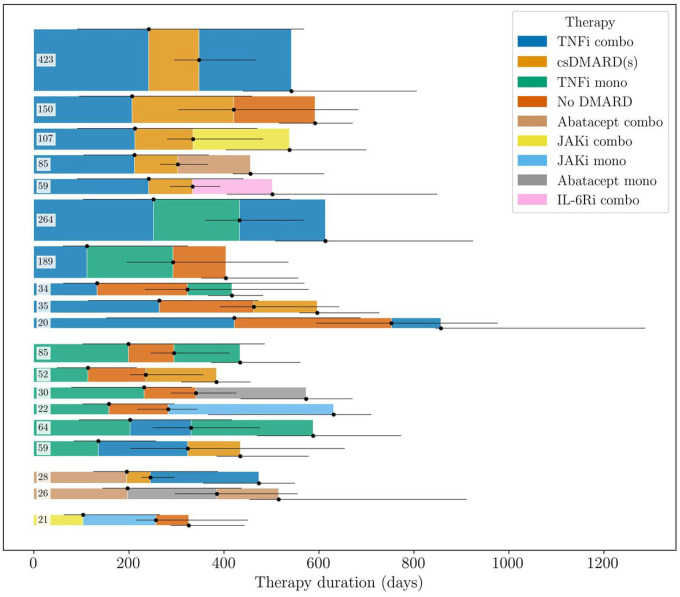
The most common treatment patterns of length three. Note that some of the patients may have been treated with additional therapies after the third therapy. The numbers indicate how many patients were treated according to each pattern. Only patterns that occurred at least 20 times in the data are shown. The height of the sequences corresponds to the number of observations of each pattern, and the length of the segments to the median therapy duration in the data. The horizontal black lines show the interquartile range (Q1, Q3) of the therapy durations. Two sequences involving therapies classified as “Other” were excluded to enhance readability.

For sequences of six therapies or more, almost all observed sequences were unique (Fig. 2). We therefore focused on sequence lengths of three to five, for which there were some recurrent patterns. Figure 3 shows the most common patterns of length three; patterns of length four and five are provided as **Supplementary Figure S1 and S2**. In total, 2615 patients (43% of the patients in the selected cohort) were treated with at least three therapies. In the two main groups of patients starting a TNFi combination therapy and a TNFi monotherapy, TNFi removal was the most common first intervention, leading to a csDMARD-only and a no DMARD therapy. In the next step, most patients restarted a TNFi, but some switched to another b/tsDMARD—mainly JAKi or abatacept. Specifically, 508 patients (19%) followed the patterns “TNFi combo → csDMARD(s) → TNFi combo” and “TNFi mono → No DMARD → TNFi mono”, while 361 patients (14%) started in the same way but instead used a non-TNFi b/tsDMARD therapy in the third line.

The strategy of restarting a therapy from a previously used therapy class was defined as therapy cycling. We observed this pattern not only in Fig. 3 but also for longer sequences (see **Supplementary Figure S1 and S2**). Table 2 shows the percentage distribution of patients by the number of restarted therapies and the number of therapies in sequence. For the first three therapies, most patients (59.5%) tried a new medication in each line of therapy. However, for sequences of four and five therapies, the majority of the patients returned to at least one previous treatment during the course of medication. Two-therapy cyclers were a specific group of returners who switched between only two distinct therapies. We see that 40.5% of the patients restarted their first b/tsDMARD therapy in the third line of therapy. For longer sequences, the percentage of two-therapy cyclers drops below 20% and 10%, respectively.

### Therapy duration over time

Figure 1 shows that the distribution of first-line therapies shifted over time. In Table 3, we present the duration, given in days, of the first three lines of therapy for different time periods in the last decade. We report median therapy duration and interquartile range. As we can see, the median duration of each of the first three therapies decreased during the study period. We also compared the therapy duration distributions within each line of therapy. We found that the difference between the medians of all pairwise distributions for 2015–2017 and 2018–2012 was non-zero with statistical significance (*p* < 0.001).

## Discussion

The goal of this work was to provide an overview of common patterns in the sequential treatment of RA starting with the first b/tsDMARD. Most patients began with a TNFi therapy, although we observed a recent shift towards JAKi therapies over the decade-long study period (2012–2021). While the choice of first-line therapy was near-deterministic as a TNFi, there was substantial variation in subsequent treatment selections, leading to many distinct treatment patterns. We identified the most common sequences of up to four therapy changes and found that therapy cycling (restarting a therapy from a previously used therapy class) was a frequent pattern. We also found that the average duration of the first three therapies decreased over the study period. We did not provide information on disease activity or adverse events for the observed patterns, and the most common patterns are not necessarily the recommended best practices. Nevertheless, identifying frequent patterns in current treatment of RA is an important step toward developing and evaluating new treatment strategies.

Real-world observational data provides a unique view of patients’ response to treatments and could be used to identify the effectiveness of different sequences of therapies. However, conducting such a study requires a quantitative understanding of current practice. For example, which patterns do we see often enough to evaluate retrospectively? First, in the current set of analyses, we found that sequences that *do not* start with an initial TNFi therapy were rare. Evaluating such patterns retrospectively would require very large data sets of patients to arrive at statistically sound results. Second, we found large practice variation in longer therapy sequences which may be exploited to identify successful strategies that deviate from current guidelines. By providing an overview of current practice, this study takes a first step in advancing RA treatment. The next steps include identifying strategies for sequential treatment with sufficient support in observed data, estimating the historical propensity for following these strategies, and adjusting for selection bias to compare their value over current guidelines.

Patients with difficult-to-treat RA (22) often undergo multiple therapies in search of a working medication. Acquiring a better knowledge of successful therapy sequences would be particularly beneficial for this group of patients. Recent work has indicated that many non-responders eventually benefit from a fourth line therapy (13), but there is little evidence of which of these “extended” sequences may work better than others. We suspect that these patients account for many of the observed patterns in this work. However, most of these patterns are not shown in our sequence visualizations in Fig. 3 because they are too rare. To draw conclusions about patients with difficult-to-treat RA and evaluate different treatment strategies for these patients, one would need to make additional assumptions and group similar sequences together. For example, patients who have tried the same *set* of therapies, with similar responses, may be comparable even if the *order in* which they tried the therapies differ.

A finding of our work is that the use of JAKi as first-line therapy increased in recent years. This trend can partially be explained by the increasing availability of these drugs. Another finding is that the duration of the three initial therapies decreased in the last decade. A possible explanation for this result is that the number of available treatment options greatly increased during this time period. An alternative explanation could be that within-class cycling, i.e., switching between drugs with the same mechanism of action, was more common in the past. However, when studying the number of within-class switches for patients who started with a TNFi combination therapy, we found no clear support for that theory. Finally, the number of outliers, e.g., patients who stayed on their first therapy for several years before they suddenly changed therapy, is naturally higher in the first interval and skews those distributions.

The trend of decreasing therapy duration was observed also in recent work by Mease et al. (23), although they used data of patients enrolled in the CorEvitas RA registry between 2004 and 2015 and studied a smaller set of therapies. There exist some studies that have tried to describe sequential therapies using Sankey diagrams (12, 13). Our sequence visualizations contain more information in the sense that they show entire therapy trajectories and not only transitions between consecutive therapies. Still, it is worth noting that Zhao et al. (13) identified the transition between TNFi and rituximab as the second most common transition between the first two lines of treatment. In contrast, there are no sequences starting in this way in Fig. 3. This may reflect differences in typical care by country (UK versus US).

The primary strengths of this study are the focus on sequential therapies and the use of a large real-world dataset from the CorEvitas RA registry. There are also limitations, for example that we did not place any restrictions on the regularity of the registry visits. As well, the median duration of follow-up in the CorEvitas RA registry is 3.4 years, which limits the amount of data for patients treated with many sequential therapies. We expect the variation in patterns to be even greater with increased duration of follow-up. Further, biologic data was not included in the current analyses, limiting the ability to understand the pathobiology of difficult-to-treat RA. The fact that we did not distinguish between individual csDMARDs may also be considered a limitation. We chose this approach to reduce the number of therapy permutations and reinforce patterns of b/tsDMARD treatment strategies—the focus of our work. For the same reason, we did not consider switching between drugs from the same class as a change of therapy, preventing such transitions from appearing in our patterns. Finally, we included only subsequences starting with the first b/tsDMARD prescription in our analysis. Most patients were treated with an initial csDMARD therapy before starting their first b/tsDMARD and not distinguishing patients based on this information may be considered a limitation. Understanding the effects of early exploration on the therapy decisions that follow is an important challenge for future work.

## Conclusions

Understanding current treatment practice is an important step in finding optimal treatment strategies. Based on data from the CorEvitas RA registry, the choice of first-line therapy is near-deterministic, but substantial variety in later-line therapy selections lead to many distinct treatment patterns. Therapy cycling is one of few patterns that are relatively common for longer sequences of therapies. Over the past decade, the duration of each of the first three therapies significantly decreased. On the one hand, the vast heterogeneity in treatment patterns indicates substantial practice variation which allows observational data to be used to evaluate new treatment strategies for RA. On the other, it brings statistical challenges to the evaluation process.

## Figures and Tables

**Table 1:** Baseline characteristics of patients in the selected cohort. N: number of patients with non-missing data; n: number of patients with baseline demographic or clinical characteristic; Q1: first quantile; Q3: third quantile; BMI: body mass index; RF: rheumatoid factor; CCP: cyclic citrullinated peptide antibody; CDAI: clinical disease activity index; DAS: disease activity score.

Characteristic	*N*	Statistics
Age, median (Q1, Q3), years	5784	58 (49, 67)
Female, *n* (%)	5784	4450 (76.94)
Race, *n* (%)	5719	
White		4539 (79.37)
Hispanic		525 (9.18)
Black		448 (7.83)
Asian		115 (2.01)
Other		92 (1.61)
Final education, n(%)	5577	
Primary school		149 (2.67)
High school		2190 (39.27)
College		3195 (57.29)
Health insurance, *n* (%)	5784	
Private		4089 (70.70)
Medicare		1874 (32.40)
Medicaid		382 (6.60)
None		115 (1.99)
BMI, median (Q1, Q3)	5653	29.19 (25.05, 34.69)
Smoker, *n* (%)	4993	859 (17.20)
Work status, *n* (%)	5656	
Full time		2362 (41.76)
Part time		472 (8.35)
Work at home		491 (868)
Student		74 (1.31)
Disabled		611 (10.80)
Retired		1646 (29.10)
Disease duration, median (Q1, Q3), years	5707	3 (1, 8)
RF positive, *n* (%)	1427	858 (60.13)
CCP positive, *n* (%)	1372	775 (56.49)
Comorbidities, *n* (%)		
History of cardiovascular disease^a^	5784	378 (6.54)
History of cancer^b^	5784	376 (6.50)
Hypertension	5784	832 (14.38)
Hyperlipidemia	5782	427 (7.38)
Diabetes	5782	316 (5.47)
Anxiety	5772	1128 (19.54)
Depression	5322	430 (8.08)
Serious infections	5772	79 (1.32)
Disease activity		
Tender joint count, median (Q1, Q3)	5713	4 (1, 10)
Swollen joint count, median (Q1, Q3)	5714	3 (0, 8)
CDAI, median (Q1, Q3)	5676	17.00 (8.88, 27.20)
DAS, median (Q1, Q3)	3303	4.15 (2.99, 5.25)
Patient self-assessment		
Pain, median (Q1, Q3)	5760	50 (20, 70)
Fatigue, median (Q1, Q3)	5730	50 (20, 75)
Morning stiffness, *n* (%)	5706	4845 (84.91)

a Coronary heart disease, stroke, TIA, carotid artery disease, peripheral arterial disease, deep vein thrombosis, pulmonary embolism, heart attack.

b Breast cancer, lung cancer, lymphoma, melanoma, other types of cancer.

**Table 2:** Percentage distribution of patients by number of restarted therapies and number of therapies in sequence. Note, by our definition, the second therapy must be different from the first therapy, so for a sequence of the first three therapies, only one restart is possible. The values in the rightmost non-empty cells indicate the percentage of two-therapy cyclers.

Number of therapies in sequence	Patient distribution over the number of restarted therapies, percent			
	0 restarts	1 restart	2 restarts	3 restarts
3	59.5	40.5	-	-
4	32.6	49.5	17.9	-
5	14.7	41.7	35.4	8.2

**Table 3:** Duration of the first three lines of therapy for different periods in the last decade. Median duration and the interquartile range are reported. Note that the duration of each line of therapy decreased during the study period.

Line of therapy	Median (interquartile range) therapy duration over time, days		
	2012–2014	2015–2017	2018–2021
1	215 (98, 518)	208 (92, 486)	153 (75, 309)
2	149 (64, 341)	150 (64, 333)	108 (61, 196)
3	172 (79, 385)	151 (71, 334)	117 (61, 242)

## Data Availability

Data are available from CorEvitas, LLC through a commercial subscription agreement and are not publicly available. No additional data are available from the authors.

## Key Messages

- Understanding current treatment practice is an important step in finding optimal treatment strategies.
- Substantial variety in later-line therapy selections lead to many distinct treatment patterns.
- This practice variation allows evaluating new treatment strategies using observational data while presenting statistical challenges.
